# Noise-Induced Min Phenotypes in E. coli

**DOI:** 10.1371/journal.pcbi.0020080

**Published:** 2006-06-30

**Authors:** David Fange, Johan Elf

**Affiliations:** 1 Department of Cell and Molecular Biology, Biomedical Centre, Uppsala University, Uppsala, Sweden; 2 Department of Chemistry and Chemical Biology, Harvard University, Cambridge, Massachusetts, United States of America; National Centre for Biological Sciences, India

## Abstract

The spatiotemporal oscillations of the Escherichia coli proteins MinD and MinE direct cell division to the region between the chromosomes. Several quantitative models of the Min system have been suggested before, but no one of them accounts for the behavior of all documented mutant phenotypes. We analyzed the stochastic reaction-diffusion kinetics of the Min proteins for several E. coli mutants and compared the results to the corresponding deterministic mean-field description. We found that wild-type (wt) and filamentous *(ftsZ^ −^)* cells are well characterized by the mean-field model, but that a stochastic model is necessary to account for several of the characteristics of the spherical *(rodA^−^)* and phospathedylethanolamide-deficient (PE^−^) phenotypes. For spherical cells, the mean-field model is bistable, and the system can get trapped in a non-oscillatory state. However, when the intrinsic noise is considered, only the experimentally observed oscillatory behavior remains. The stochastic model also reproduces the change in oscillation directions observed in the spherical phenotype and the occasional gliding of the MinD region along the inner membrane. For the PE^−^ mutant, the stochastic model explains the appearance of randomly localized and dense MinD clusters as a nucleation phenomenon, in which the stochastic kinetics at low copy number causes local discharges of the high MinD^ATP^ to MinD^ADP^ potential. We find that a simple five-reaction model of the Min system can explain all documented Min phenotypes, if stochastic kinetics and three-dimensional diffusion are accounted for. Our results emphasize that local copy number fluctuation may result in phenotypic differences although the total number of molecules of the relevant species is high.

## Synopsis

Many molecules inside a living cell do not have time to diffuse through the whole cell in-between reactions. Furthermore, the chemical reactions are random and discrete events. In this study, the authors study an example in which these aspects of intracellular chemistry need to be considered when we try to understand how a biological system works.

The authors have investigated the spatial oscillation patterns that are displayed by the Min system of Escherichia coli. In wild-type *E. coli,* the Min proteins oscillate back and forth between the cell poles to help the bacterium find its middle before cell division. The authors used computer simulations to explain why the oscillation patterns change the way they do in different mutants of E. coli. They find that two of the mutant phenotypes can only be explained if one considers the randomness and discreteness of chemical reactions in addition to the spatial characteristics of the process. Particularly interesting is the phospathedylethanolamide-deficient phenotype, in which large dense clusters of MinD protein appear for some time at random locations on the membrane. The authors believe that this phenotype is due to a nucleation phenomenon, in which the stochastic kinetics at low copy number is amplified to macroscopic proportions.

## Introduction

**Figure 1:**
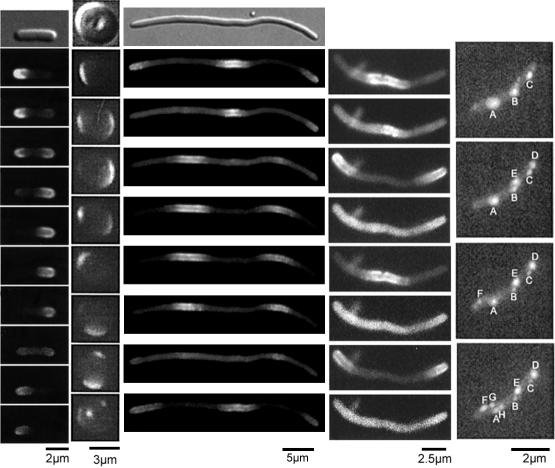
Time-Lapse Microscopy Images of GFP-Tagged MinD in Four Different E. coli Strains From top to bottom: time evolution. From left to right: wt, spherical *rodA* ^−^ *,* filamentous *ftsZ* ^−^ (long), filamentous *ftsZ* ^−^ (short), PE^−^ E. coli. Images are reproduced from (left to right) from the following references: [21], [25], [27], [15], and [26].

**Figure 2:**
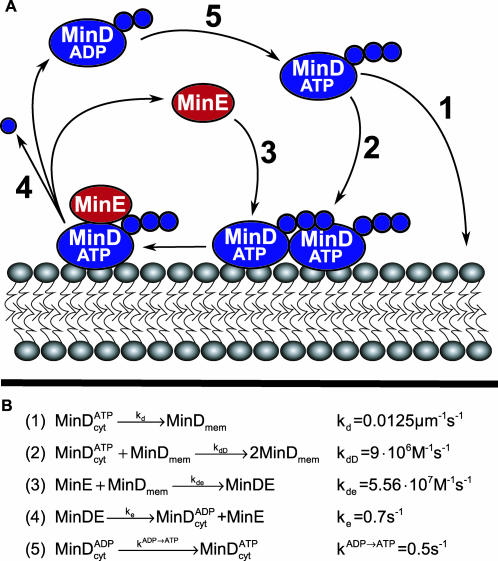
Cartoon of the Min System and the Corresponding Reaction Scheme and Rate Constants (A) Shows the Min system and (B) shows the corresponding reaction scheme and rate constants. The reaction scheme is essentially adapted from Huang et al. [30]. The diffusion constant for proteins in the cytoplasm is 2.5 × 10^−8^ cm^2^s^−1^ and in the membrane, 1 × 10^−10^ cm^2^s^−1^. In the PE^−^ mutant: *k_d_* = 1.25 × 10^−6^ μms^−1^ and the membrane diffusion is 1 × 10^−12^ cm^2^s^−1^.

Quantitative modeling of biological processes is becoming increasingly important as the processes we seek to understand become more and more complicated. The necessity for quantitative modeling is especially compelling when the process of interest displays spatiotemporal pattern formation, such as the oscillations of the Min proteins seen in Figure 1. In this case, it is obvious that the cartoon representation of the possible reactions, e.g., that in Figure 2, does not give all the information about the system. It is not apparent from the cartoon whether the reactions can give rise to the observed phenotype or if the cartoon is overly complicated in relation to what is required to reproduce the experimentally observed patterns. In order to investigate the requirements for the observed dynamical behavior, a quantitative model is necessary.

The Min system, which directs E. coli cell division to the middle of the cell [1], is an extraordinary example of how quantitative modeling has helped to clarify spatiotemporal pattern formation of biological relevance [2]. In this study, we have used a stochastic reaction-diffusion model in three spatial dimensions to study how mutant Min phenotypes arise due to variations in the geometry of the bacterial cell or its kinetic parameters. The stochastic approach is motivated by previous studies of how the intrinsic chemical fluctuations in spatially extended systems can cause radically different properties than what would be described by a mean-field model. Molecule discreteness and fluctuation in non-homogeneous systems has, for instance, been shown to create new steady states [3], drive spatial oscillations [4], cause spatial phase separation of a bistable system [5], or drive the irregular relocation dynamics of Soj protein in Bacillus subtilis [6].

However, from the recent stochastic analysis of the Min system in three dimensions [7,8], it appears that the deterministic mean-field picture of wild-type (wt) E. coli does not change much when the chemical fluctuations are considered. Our results confirm these observations, but they also show that molecular discreteness and spatially localized fluctuations are likely to cause the phenotypic characteristics of the *rodA* ^−^ and phospathedylethanolamide-deficient (PE^−^) E. coli mutants.

### The Min System in E. coli

The Min system consists of the MinC, MinD, and MinE proteins expressed from the *minB* operon [1]. Together with the nucleoid occlusion system, which excludes cell division over a chromosome, the Min system facilitates accurate positioning of the septum at mid-cell before cell division in E. coli [9]. The Min proteins prevent septum formation near the cell pole by inhibiting FtsZ polymerization. Polymerization of the FtsZ protein into the Z-ring is the first step in septum formation [10]. The polymerization is inhibited by MinC in vitro, and in vivo the *minC* mutants display the minicell phenotype [11]. In this phenotype, many cells lack a chromosome after cell division [1] because the septum has formed in the polar region of the cell. In vivo, MinC co-localizes with MinD [12,13], which is oscillating from pole to pole with a frequency of about one per minute (see Figure 1). The growing and shrinking polar zones of MinD and MinC thus exclude the FtsZ ring formation in the polar regions, which directs it to mid-cell.

In order to understand what drives the oscillation, the components of the system have been biochemically characterized in some detail. MinD in association with ATP binds cooperatively to the membrane independently of the presence of MinE [14]. However, the oscillation of MinD is dependent on MinE. If MinE is absent, MinD will be distributed evenly over the cell membrane [15,16]. MinE binds membrane-associated MinD and induces ATP hydrolysis, which results in release of both MinD and MinE from the membrane [14,17–19]. In the cell, MinE forms a ring-like structure at the rim of the shrinking MinD polar zone. When all MinD has been driven from the old pole, the MinE ring reassembles at mid-cell, on the rim of the new MinD zone [20,21]. The MinE oscillations are dependent on MinD. If MinD is absent, MinE is homogeneously distributed in the cytoplasm [22].

In addition to wt *E. coli,* three different mutants with interesting Min phenotypes have been described. In the filamentous *ftsZ^ −^* mutant, MinD forms an oscillating striped pattern with a period slightly longer than the maximal length of the wild type E. coli (~4 μm) (Figure 1) and unaltered oscillation frequency [13,15,20,23]. In *rodA* ^−^ mutants with a spherical phenotype, the oscillation sometimes changes direction (Figure 1). Furthermore, *rodA* ^−^ mutants have a more diffuse localization of the MinE protein than the rod-like phenotypes [24,25]. In the PE^−^ strain, MinD is localized in tight clusters (spots), which randomly appear and disappear at a minute timescale (Figure 1) [26].

The first quantitative models of the Min system were developed at the same time by Meinhardt and de Boer [27], Howard et al. [28], and Kruse [29]. These initial studies have been followed by several others over the last few years, e.g., [4,7,8,23,30–33]. All the quantitative models have the interaction between MinD, MinE, and the membrane in common. The membrane-bound molecules have slower diffusion rates [29] or are non-diffusing [27,28,30,31]. The MinD protein binds to the membrane, followed by a subsequent binding of MinE to membrane-bound MinD [27,29–31], or MinDE complexes bind to the membrane [28]. Furthermore, the cooperativity in binding of MinD to the membrane is treated differently in the different models, as is MinD's change from ADP to ATP form. Most models deal with the oscillations in wt and filamentous cells, but Huang et al. [32] has also applied their mean-field model to spherical cells *(rodA^−^)*. Recently, results from two stochastic three-dimensional (3D) simulations of the Min oscillations in wt E. coli were published [7,8]. These simulations show that the mean-field model of wt E. coli by Haung et al. [30] works well also when stochasticity is considered. In the present work, we go one step further and show that a stochastic model is necessary to account for the characteristic properties of the phenotypes in *rodA^−^* and PE^−^ strains.

### Stochastic and Mean-Field Modeling of Reaction–Diffusion Systems

Chemical reactions are stochastic events, meaning that it is not possible to know when and where the next reaction will occur. The probabilities for the reaction events can, however, be modeled, and the time evolution of the system can therefore be described probabilistically. In this article, we model the stochastic reaction–diffusion kinetics of the Min systems using the framework provided by the reaction–diffusion master equation (RDME) [34–37]. The relation to the complementary Smoluchowski framework [38–42] for modeling stochastic reaction diffusion kinetics is described in the Materials and Methods section.

In the RDME description, the total system volume is divided into a large number of small subvolumes (25^3^–50^3^ nm^3^ in our model). The number of molecules of the different species in the different subvolumes describes the state of the system. The state changes when the molecules in any subvolume react or when a molecule diffuses between subvolumes. The RDME provides the probability distributions for these different events.

We compare the stochastic time evolution of the Min system with the corresponding mean-field approximation. The approximation is that the state, i.e., the number of molecules in different subvolumes, changes with the average rate at each point in time.

A more informative description of the difference between the stochastic and the mean-field approach is given in the Materials and Methods section.

## Results

### The MinD MinE Model

The elementary interactions between the different forms of the MinD and MinE proteins are described in Figure 2. In the cytoplasm, MinD can be in either ATP or ADP form (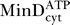 or 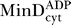 ), and on the membrane, either free in ATP form (MinD_mem_) or bound in complex with MinE (MinDE). MinE is either freely diffusing in the cytoplasm (MinE) or in complex with MinD on the membrane (MinDE). 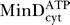 can bind to the membrane either spontaneously (reaction 1) or by being recruited by membrane-associated MinD_mem_ (reaction 2). MinD_mem_ also recruits cytosolic MinE (reaction 3). MinD-associated MinE hydrolyzes the ATP on MinD_mem_, which results in the release of 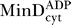 and MinE from the membrane (reaction 4). In the cytoplasm, the ADP of 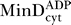 is exchanged for ATP (reaction 5). The reaction scheme is adopted from Huang et al. [30]. We have, however, removed the association of 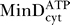 to MinDE from the original scheme since we could not find biochemical support for this reaction. The cytosolic proteins are given the diffusion rate constant of 2.5 × 10^−8^ cm^2^s^−1^ [30], and for the membrane-bound proteins we use 1.0 × 10^−10^ cm^2^s^−1^. (See the discussion about membrane diffusion and polymerization in the Materials and Methods section.)

The system was simulated in three different geometries corresponding to wt, filamentous, and spherical E. coli. The wt geometry is defined by a cylinder of length 3.5 μm with half-sphere caps of radius 0.5 μm. The geometry of the filamentous mutants [15] is like the wt except for the length of the cylinder, which is 9.5 μm or 14.5 μm. The spherical mutant [25] is modeled as a sphere with a radius of 1.5 μm. In all geometries, the initial concentrations are 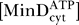 = 0.818 μM, [MinD_mem_] = 0, 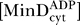 = 0.818 μM, [MinDE] = 0, and [MinE] = 0.425 μM. For the wt geometry, this corresponds to 4,002 MinD and 1,040 MinE molecules, which is in agreement with experimental estimates [43]. In the stochastic simulations, the MinD and MinE proteins are distributed randomly throughout the cytoplasm, corresponding to a homogeneous initial distribution. In the mean-field simulation, the system needs a non-homogeneous initial distribution to do anything at all, therefore 3/4 of the molecules are initially located in the cytoplasm of one half of the cell and 1/4 in the other half, unless otherwise stated.

The reactions rates used are presented in Figure 2B. The values for these five parameters are basically unknown both in vivo and in vitro. Our reaction rates were therefore obtained by extensive parameter variations over a large parameter space (~100-fold variations in each dimension) aimed at making the oscillation of the wt and filamentous cells as similar as possible to the experiments (Figure 1). The parameters that we found optimal for wt and filamentous cells were tested by the model's ability to reproduce the phenotype of the *rodA^−^* mutant, after changing only the cell geometry to a sphere. The alteration of the membrane composition in the PE^−^ strain does, however, interfere with the Min systems' membrane interaction, and the parameters for MinD's interaction with the membrane were changed to accommodate this mutation (see section about PE^−^ below.)

The exact model descriptions, i.e., the SBML files that were used to make the simulations, are supplied in Datasets (Dataset S1–S9). The 3D geometries are discretized into cubic subvolumes with side length 50 nm, which corresponds to 32,500, 78,500, or 113,000 subvolumes for wt, filamentous (10 μm), and spherical cells, respectively. Tests done with smaller and larger subvolumes (side lengths 25 nm or 100 nm) did not display any significant differences. Simulating one oscillation of the wt cell using MesoRD ([44], Materials and Methods) including 3.5 × 10^8^ events takes 25 min on a Intel Xeon 3.06 GHz and requires 20 Mb of RAM.

### Pattern Formation in the Different Min Mutants

**Figure 3:**
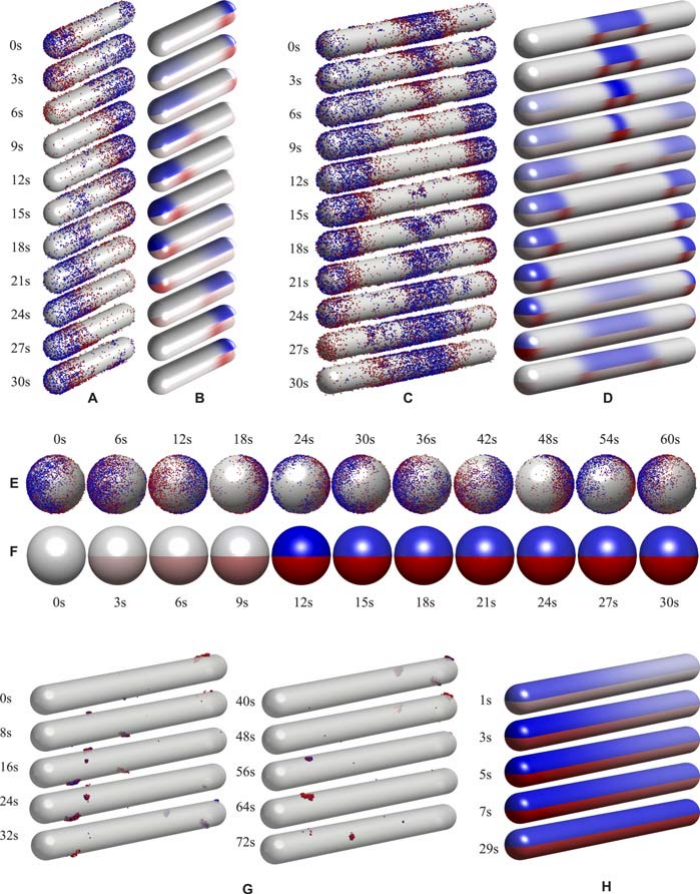
Comparison of Stochastic and Mean-Field Simulations in wt E. coli Cells and Three Different Mutant E. coli Strains The wt geometry (A) and (B), filamentous geometry *(ftsZ^ −^)* (C) and (D), spherical geometry *(rodA^−^)* (E) and (F), and PE^−^ cells with filamentous geometry (G) and (H) are shown. (A), (C), (E), and (G) show the stochastic simulations and (B), (D), (F), and (H) show the mean-field simulations. Membrane-bound MinD is shown in blue, and MinE in complex with MinD on the membrane is shown in red. The cells in (B), (D), (F), and (H) have been divided into two halves (upper and lower) to show both the MinD and the MinE concentration fields in the same plot. The discretized solution of the mean-field simulations has been mapped onto a smooth surface to facilitate visualization. In (G), the cell surface is transparent to allow visualization of the clusters on the back.

Figure 3 shows comparisons of stochastic and mean-field simulations for the different E. coli mutants; the corresponding movies (Videos S1–S9) do, however, give a much better understanding of the systems' dynamics. For the stochastic simulations, the molecules are visualized as small spheres, and for the mean-field simulations, the concentration fields are visualized with a spatially continuous color code, in which higher intensity corresponds to higher concentration.

#### The wt cell.

The first thing to notice for the wt cells in Figure 3 is that both the stochastic and the mean-field models oscillate nicely when including only the five reactions in Figure 2B. Furthermore, there are no large differences between the stochastic and deterministic descriptions, i.e., fluctuations do not significantly change or contribute to the properties of the wt system. This observation is in agreement with recent stochastic simulations of wt cells by Kerr et al. [7] and Pavin et al. [8]

The wt geometry shows MinD oscillating between the poles, followed by a narrow MinE ring. In agreement with experiments, both the stochastic and the mean-field models display clear growth of membrane-associated MinD zones from the poles to mid-cell and shrinkage back again. The growth phase is fast and due to rapid recruitment of the abundant 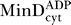 from the cytoplasm. The shrinkage phase is induced by attachment of free MinE to the rim of the new zone. The rate of shrinkage depends on the amount of MinE and how rapidly it hydrolyzes the membrane-bound MinD^ATP^. Thus, if the concentration of MinE is increased, the oscillation frequency also increases. In the wt simulations, the average period times are 53, 40, 31, 26, and 22 s for 0.8, 0.9, 1.0, 1.1, and 1.2 times variation in the concentration of MinE, respectively. The corresponding numbers for variations in MinD concentration are 20, 25, 31, 38, and 48 s for 0.8, 0.9, 1.0, 1.1, and 1.2 times variation in the concentration of MinD. These responses to variations in MinD or MinE correspond to what has been observed experimentally [15] and to what has earlier been described for the mean-field system [30].

**Figure 4:**
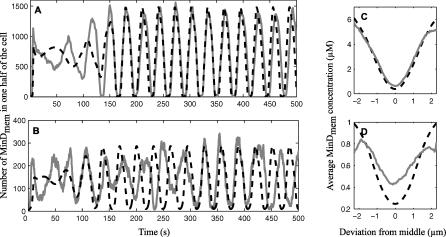
Comparison of Stochastic and Deterministic Simulations at Different Concentrations of MinD and MinE in a wt Cell The number of membrane-bound MinD molecules in one half of a cell for wt (A) and 25% of wt concentration (B). Time-averaged localization of MinD for stochastic and deterministic simulation for wt concentration (C) and for 25% of wt concentration (D). Stochastic simulations are show in solid gray, and deterministic simulations in dashed black.

Although the stochastic properties are not prominent in the wt system, some predictions can only be made using the stochastic description. For instance we estimate that the standard deviation in period time divided by the mean is 2.75%. This phase drift is more pronounced for a smaller number of molecules. When the concentrations of MinD and MinE are simultaneously reduced by 50% or 67%, the phase drift is increased by 4.5% and 6.9%, respectively. In Figure 4A and 4B, it is demonstrated that the oscillation of the stochastic model becomes aperiodic when the concentrations are reduced by 75%, whereas the oscillations are unaltered in the corresponding deterministic model.

Since one physiological role of the MinD protein is to keep the co-localized protein MinC away from mid-cell, one may also be interested in how the MinD protein is distributed over the length of the cell and how this distribution depends on the copy numbers. In Figure 4C, we see the average MinD concentration over 25 oscillations for the wt cell. This is compared to the case in which the concentrations of MinD and MinE are reduced by 75% (Figure 4D). The increased influence of noise at lower copy number makes the dip of MinD at mid-cell less pronounced, as was already concluded in earlier studies [4,7].

#### The filamentous *ftsZ* ^−^ cell.

**Figure 5:**
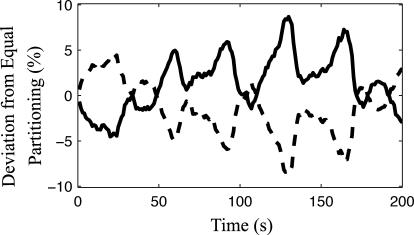
Variation in the Number of MinD Proteins in the Two Halves of the *ftsZ^ −^* 10-μm Cell in the Stochastic Model Percentage of deviation from equal partitioning (i.e., 50% of the total molecule number goes to each pole) of membrane-bound MinD molecules. The percentage of deviation for one half of the cell is plotted as solid black and the percentage of deviation in for other half is plotted as dashed black.

The filamentous 10-μm E. coli falls into a doubled pattern, with MinD cycling between mid-cell and the poles (compare with Figure 1), closely followed by MinE rings. When the filamentous cell is made longer (15 μm), it falls into the tripled pattern as seen in experiments on longer cells (see Figure S1 and Video S7). As for wt, there are no qualitative differences between the stochastic and mean-field models. The stochastic model does, however, predict the variability in the number of MinD that goes into each stripe. For the 10-μm filamentous cell 48.8 ± 4.8% (average ± standard deviation) of the proteins go to the respective cell pole. This variation is much greater than what one would get under the assumption that each protein goes to a random stripe, which would give a binomial partitioning with 50 ± 0.5% of the proteins at each pole. The additional variability is due to the small variation in time when a new set of stripes is formed. The stripe that is formed first will rapidly recruit a disproportionately higher fraction of the MinD proteins. In Figure 5, the variability in the number of MinD proteins that go to respective cell poles is illustrated for a few oscillations.

#### The spherical *rodA* ^−^ cell.

**Figure 6:**
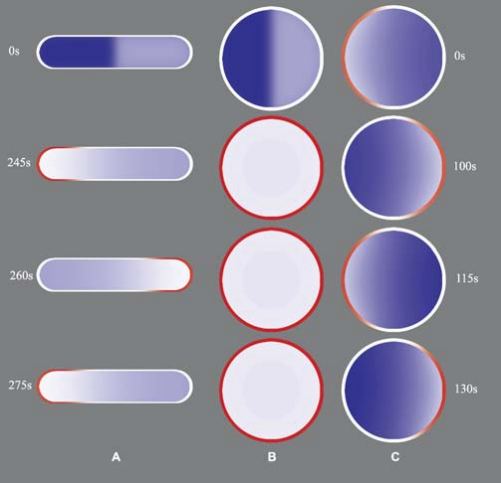
Comparison of Mean-Field Results for wt and Two Spherical Cells with Different Initial Conditions Membrane-bound MinD is shown in red, and cytosolic MinD^ATP^ is shown in blue. The color intensity is proportional to the integrated concentration of molecules along the axis perpendicular to the projection plane. In (A) (wt) and (B) (spherical), the series are initialized with 3/4 of the MinD in the cytoplasm in one half of the cell and 1/4 in the other half. In (C) (spherical), the series is initialized with most of the MinD membrane bound at one side of the cell.

For wt and filamentous *E. coli,* the stochastic model mainly justifies the more analytically accessible mean-field description. However, for the *rodA^−^* and PE^−^ mutants, the stochastic models explain experimental observations that cannot be accounted for by mean-field models. Starting with the spherical *rodA* ^−^ mutant in Figure 3E and 3F, it seems like only the stochastic model of the spherical cell oscillates. The figure is, however, slightly misleading, as the mean-field model can be made oscillatory with other initial conditions. The dependency of initial conditions is described further in Figure 6. Here, we see the concentrations of 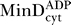 and 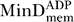 in a cross-section of the wt and spherical cells at different points in time. For the spherical cell, two different initial molecule distributions are used. When 3/4 of the MinD molecules are initially located in the cytoplasm in one half of the spherical cell, as in Figure 3F, the molecules spread out evenly, and no oscillations are initiated. In contrast, if all the MinD molecules are bound to the membrane in one half of the cell, the oscillations start immediately (Video S6). Thus, the mean-field model of the spherical cell is bistable; for some initial conditions, it falls into a stable point attractor and stays there, and for others, it falls into a limit cycle attractor.

**Figure 7:**
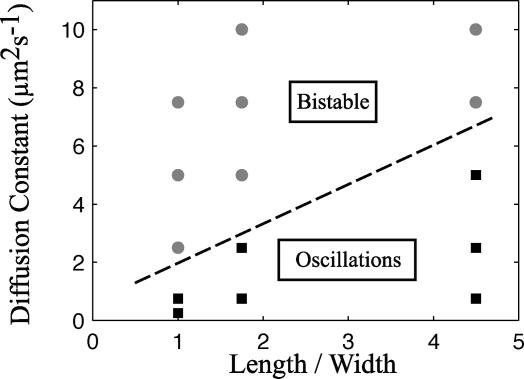
Bistability Diagram for Different Diffusion Constants and Cell Geometries The mean-field model was solved for three different geometries: Spherical mutant (length/width = 1), wt (length/width = 4.5), and an intermediate geometry (length/width = 1.75; cylinder of radius 1 μm and length 1.5 μm padded with half spheres of radius 1 μm). The combinations indicated with black squares display oscillations when started from the initial condition in which 3/4 of the MinD molecules are started in one half of the cell and 1/4 started in the other. The gray circles represent combinations that display bistability: When the simulation is started from the initial condition described above, the system will go to a stationary homogeneous state. When the simulation is started from an existing MinD accumulation at one of the poles, it will start oscillating.

The requirements for bistability are further characterized in Figure 7 **,** where we have varied the cell shape and the intracellular diffusion constant independently to determine under which conditions the mean-field model is bistable. We conclude that more-elongated cells require higher rates of intracellular diffusion to display bistability. The spherical cell is bistable if the intracellular diffusion rate is faster than 5 × 10^−9^ cm^2^s^−1^ whereas the rod-shaped 4.5-μm wt cell is bistable for diffusion rate constants over 5 × 10^−8^ cm^2^s^−1^. It seems like a fast redistribution of molecules in the cytoplasm prevents formation of sufficiently large local membrane occupancy to initiate cooperative recruitment of MinD and thereby start the oscillations. When the fluctuations are included in the model, the potential non-oscillatory state is not a problem since a few spontaneously associated MinD molecules are sufficient to initiate the oscillations (see the PE^−^ phenotype below).

The stochastic model also suggests a simple explanation for the change in oscillation direction that is seen experimentally in spherical cells (Figure 1) [25,45]. Since the spherical cell is symmetric, there is no preferred direction of oscillation, and the stochastic model can change direction relatively freely. This is also true if the cell is only nearly spherical. There is, however, a preference for forming the new MinD zone approximately opposite to the old MinD zone, because the concentration of 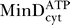 is highest there. In a mean-field model of an oscillating spherical cell, the direction of oscillation is dictated by the initial condition (Figure 6C) or, if the cell is only nearly spherical, by the direction of its long axis, as was reported in the mean-field characterization of the spherical phenotype by Huang and Wingreen [32].

In the stochastic model, we also initially observe that the MinD molecules are gliding around the cell membrane in a rotational motion (see Video S5) as was recently observed experimentally by Shih et al. [45].

#### The spotty PE^−^ cell.

The most interesting differences between the stochastic and mean-field descriptions are seen in the “spotty” PE^−^ phenotype. Here, there are large qualitative differences between the models, and only the stochastic model can explain the experimentally observed behavior.

To account for the abnormal MinD membrane interactions in the spotty (PE^−^) phenotype, we reduced the rate of spontaneous MinD association to the membrane and the membrane diffusion constant (see Figure 2B). Only equilibrium data for MinD binding to PE liposomes in the absence of MinE are available. Since these data do not say anything about the individual rate constants, it will be interesting to see if the reduced rate of spontaneous MinD association that we predict is found in the real system or if the model is inconclusive.

With the modified MinD membrane interaction, the stochastic model nicely reproduces the appearance and disappearance of dense MinD clusters in the PE^−^ phenotype (Figure 3G and Video S8). Both the number of spots and the frequency of their appearance are well reproduced in the stochastic model. However, in the corresponding mean-field model, the molecules spread out evenly over the cell (Figure 3H and Video S9), and no oscillations are observed.

**Figure 8:**
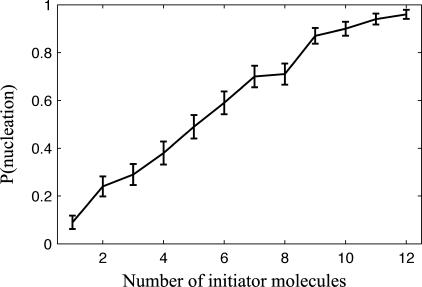
Probability for Nucleation as a Function of the Number of Initiator MinD_mem_ Molecules The stochastic model with PE^−^ parameters was solved for a box (5 μm × 1 μm × 1 μm), with membrane on one of the 1-μm × 1-μm sides. The simulations were initialized with a membrane occupancy of 1–12 MinD molecules. A total of 100 trajectories were gathered for each number (1–12) of initiator molecules. The probability of nucleation is defined as the fraction of trajectories reaching more than 100 membrane-bound MinD molecules. More detail is given in Figure S2.

To characterize the phenomenon further, we initialize the stochastic simulation with 1–12 MinD_mem_ bound to a well-defined membrane location and determine the probability that a certain number of initiator molecules will lead to the formation of a MinD spot (more than 100 membrane-bound MinD). The result is presented in Figure 8 and in more detail in Figure S2. The probability of spot formation after one MinD_mem_ has bound to the membrane is about 10%, whereas the probability of spot formation is about 90% if ten MinD_mem_ are initially bound. The critical nuclei size for which there is a 50% chance to get a spot is between five and six initiator molecules. There is no simple way to express the nucleation probabilities explicitly in terms of the rate constants since there are many paths through state space to spot formation. From Figure S2, it is further demonstrated that the membrane binding is excitable; above an activation threshold, the number of membrane-bound MinD reaches the same level independently of how many MinD that nucleated the process.

**Figure 9:**
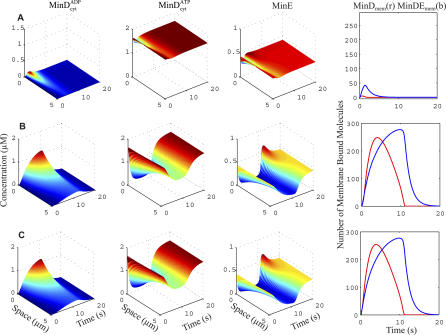
Deterministic Time Evolution for Cluster Formation in the PE^−^ Phenotype The mean-field equations with PE^−^ parameters were solved for a box (5 μm × 1 μm × 1 μm), with membrane on one of the 1-μm × 1-μm sides. The simulations were initialized with a membrane occupancy of MinD corresponding to 5, 7.5, and 10 molecules in the center of the membrane for (A), (B), and (C), respectively. The right-most column displays how the number of membrane-bound MinD (red) and MinDE (blue) complexes change in time. In the other plots, we see the concentrations of the cytosolic proteins at different distances from the membrane for different points in time.

The reason for the activation threshold and the excitability of the system is most clearly seen in a deterministic model, which can be initialized above or below the threshold. In Figure 9, we see the mean-field version of spot formation. The data have been projected down to one spatial dimension so that the evolution in time and space can be visualized simultaneously. In Figure 9A, the process is initialized below the activation threshold with five MinD_mem_ in the membrane. After initialization, some of the cytosolic MinE and MinD^ATP^ are rapidly recruited to the membrane. There is, however, always a sufficiently high local concentration of MinE close to the membrane to get a strictly decreasing concentration of membrane-bound MinD (red curve in the leftmost column of Figure 9A). The deterministic simulation visualized in Figure 9B is initiated with 7.5 membrane-bound MinD_mem_, which is above the activation threshold. In this case, a sufficiently large amount of MinD is recruited to the membrane to sequester the local MinE pool into MinDE complexes, corresponding to a saturation of the MinE activity. When the local intracellular MinE supply is depleted, there is still plenty of 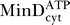 available for binding. These MinD molecules are recruited to the membrane, and a burst of membrane-bound MinD_mem_ accumulates unhindered by the saturated MinE system. After some time, the supply of 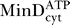 close to the membrane runs out and is replaced by a 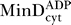 pool that cannot bind back to the membrane before nucleotide exchange. At this point, the MinE molecules take control of the situation as the rate of MinD association drops below the MinE activity at saturation. Essentially all membrane-bound MinD_mem_ proteins are hydrolyzed in this stage, which results in a burst of cytosolic 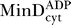 and cytosolic MinE close to the membrane. Finally, the cytosolic 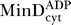 is converted back to 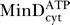 . From this point on, nothing more happens in the mean-field model. However, in the stochastic model the spontaneous binding of a 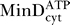 to the membrane has a small probability (10%) of nucleating the formation of a new MinD_mem_ cluster as the 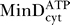 to 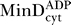 potential is restored.

In Figure 9C, the process is nucleated with 10 MinD^ATP^ on the membrane. The result is indistinguishable from the case with 7.5 initiator molecules, which demonstrates that essentially the same response is exited as soon as the activation threshold is passed.

## Discussion

We have developed computational methods (see Materials and Methods) that have made it possible to simulate a model of the Min system based on the reaction–diffusion master equation [34,36,37,46]. The stochastic reaction–diffusion model reproduces the oscillations of wild type *E. coli,* as well as the filamentous ftsZ*^−^* phenotype, the spherical rodA^−^ phenotype and the PE^−^ phenotype (Figure 1 and 3, and Videos S1–S5). The stochastic time evolutions of the systems have been compared to the corresponding deterministic mean-field simulations.

For wt *E. coli,* we reproduce the results from previous stochastic reaction–diffusion simulations [7,8]. These simulations essentially justify the previous mean-field models of wt *E. coli,* which implies that the wt oscillations are robust to the natural perturbations induced by the stochastic chemical reactions and diffusion.

In agreement with previous observations by Howard and Rutenberg [4] and Kerr et al. [7], we find that the MinD minimum at mid-cell is close to that of the deterministic model for the wt protein concentrations, but that the minimum broadens significantly if the number of molecules is reduced (Figure 4D).

Our simulations further predict a noise-induced phase drift of 2.75% per oscillation in wt cells (Figure 4A). The phase drift is predicted to increase significantly if the concentrations of MinD and MinE are decreased by more than 50% from the reported wt values. On top of the stochastic phase drift for a fixed number of MinD and MinE molecules that we describe here, one should also expect variations in oscillation period over the cell cycle and between different bacterial cells because the MinD to MinE ratio will vary due to uneven partitioning after cell division [33].

Both the mean-field and the stochastic models accurately reproduce the striped patterning in filamentous cells without adding any “topological markers.” Such markers have, in some studies, been introduced to position the MinD zones at the desired places [31]. Our observations do not rule out the presence of topological markers, but they show that topological markers are at least not necessary to get the oscillating striped pattern even when the stochastic properties of the system are considered. The stochastic model also predicts that the fraction of MinD that goes into each stripe is very unevenly distributed (Figure 5), due to the small difference in time between the formation of the different stripes.

For the parameters that we found optimal for reproducing the experimental oscillations in wt and filamentous cells, the mean-field model of the spherical cell is bistable (Figure 6). Depending on initial conditions, the spherical cell model falls into a limit cycle attractor or into a stable point attractor with a homogeneous distribution of molecules. However, when the stochastic fluctuations are considered, the system is driven away from the point attractor and into a limit cycle in a noise-induced transition [47]. It has been shown earlier that chemical noise can drive biological oscillations [4,6,48,49], but as far as we know, nobody has demonstrated that the phenomenon can also depend on the geometry of the system. The practical consequence of the noise-induced oscillations is in this case that the Min system is less sensitive to variations in cell geometry.

The formation of dense MinD clusters in the PE-lipid–deficient strain is explained here as a nucleation phenomenon. The low spontaneous 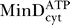 association keeps the cytosolic MinD^ATP^ solution supersaturated until the number of membrane-associated MinD reaches a threshold in which MinE-mediated hydrolysis of 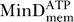 cannot keep up with MinD association, and a dense MinD cluster is formed. The threshold can only be reached through a sequence of discrete and unlikely events and is never reached in the mean-field description.

Nucleation occurs infrequently in relation to all other events in the system. It is therefore computationally demanding to accurately determine the rate at with MinD clusters are formed using brute force simulations. Inspired by the methods for Forward Flux Sampling [50,51], we can, however, make an estimate of the cluster formation rate by multiplying the rate of association of single MinD^ATP^ to the membrane in the PE^−^ model with the probability of cluster formation given that one MinD^ATP^ has bound to the membrane. The association rate is estimated by the average 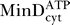 concentration times the surface area times the association rate constant, that is, 6,550 molecules/7.75 μm^3^ × 32.99 μm^2^ × 5 10^5^ μm s^−1^ = 1.39 s^−1^. The probability of cluster formation after that one MinD has bound is approximated by the probability of cluster formation if one MinD has bound to the membrane and the other molecules are randomly distributed (≈10%), as given by Figure 8. In total, the rate of cluster formation for our parameters is estimated to be 0.14 s^−1^.

With some notable exceptions ([4,5,7,33,52–56]), this study is one of the first that explores a stochastic reaction–diffusion model of a biological system. It is therefore interesting to consider which of the results from the Min system are more generally applicable.

The first lesson from the Min system is that the fluctuations can destabilize one of several stable attractors of the mean-field model and make that attractor practically unimportant for the real system. This was observed in the Min system for the round cell, in which the mean-field model has one stable fixed point and a limit cycle attractor, whereas the stochastic system only uses the limit cycle.

The second lesson is that a stochastic system can explore different parts of neutrally stable attractors, when the mean-field model will be confined to a stationary point or a limit cycle. In the stochastic model of the Min system, this was exemplified by the phase drift in the wt cell and the change in oscillation directions seen in the round cell.

The final lesson is that the discrete and probabilistic aspect of stochastic kinetics sometimes causes a sufficiently high local concentration to initiate a process with an activation threshold. This was seen in the Min systems in the formation of dense clusters after spontaneous membrane association of a few MinD molecules in the PE^−^ mutant.

As with all other stochastic phenomena in intracellular kinetics, the possibility of localized stochastic nucleation is a constraint for the wiring of intracellular reaction networks, but also a potentially useful process. It is, for instance, a constraint for signaling systems that depend on local activation of sensory systems at the membrane followed by signal amplification for efficient propagation to intracellular targets [57]. It is important that such signaling systems are not spontaneously activated by a localized stochastic nucleation of the kind we have seen for the PE^−^ mutant.

On the other hand, spatially localized, low copy number fluctuations could be used to generate variability in cell shape, for instance by nucleating the formation of morphogen clusters at random localization in the cell. Such a mechanism would be a spatial analog to the stochastically activated excitable systems that are used to generate variability in a cell population, e.g., the sporulation process in B. subtilis [58].

## Materials and Methods

### Stochastic and mean-field modeling of reaction–diffusion systems.

Chemical reactions are stochastic events, meaning that it is not possible to know when and where the next reaction will occur. The probabilities for the reaction events can, however, be modeled, and the time evolution of the system can therefore be described probabilistically. Stochastic reaction–diffusion kinetics is commonly modeled by the RDME [34,36,59]. In the RDME framework, the total system volume is divided into a large number of subvolumes. The number of molecules of the different species in the different subvolumes describes the state of the system. The subvolumes must be small enough to be homogenized by diffusion on the timescale of the chemical reactions and, at the same time, be significantly larger than the molecules themselves, such that molecules can be fully dissociated within the same subvolume and microscopic association–dissociation kinetics can be disregarded [5,41].

The number of molecules in the different subvolumes, i.e., the state of the system, changes when the molecules in any subvolume react or when a molecule diffuses between subvolumes. In the stochastic framework, the reaction and diffusion events are probabilistic, and the state changes in discrete steps when an event occurs. The probability that a certain reaction occurs in a subvolume of volume Ω during the next time interval *dt* is *dt*Ω*r*(**x** *_i_*), where it is indicated that the rate *r* of the reaction depends on the concentrations of reactants **x** *_i_* in the subvolume *i.* For instance, the rate, *r,* of the reaction 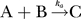 is given by *r* = *k_a_a_i_b_i_,* where *a_i_* = *n_A,i_*Ω^−1^ and *b_i_* = *n_B,i_*Ω^−1^ are the concentrations of A and B in subvolume *i,* and *k_a_* is the second-order rate constant of association. If this reaction occurs, the number of A and B molecules in subvolume *i, n_A,i_,* and *n_B,i_,* respectively, are reduced by 1 and the number of C molecules is increased by 1.

The event that a molecule diffuses to a neighboring subvolume is treated as a first-order reaction with a rate constant of *k_diff_* = *D/ℓ* ^2^ *,* where *D* is the diffusion constant for the diffusing species and *ℓ* is the side length of the subvolume. The probability that an A molecule diffuses from one subvolume to one of its neighbors in the next short time period *dt* is thus *dt*Ω*k_diff_a_i_* = *dtk_diff_n_A,i_,* where *a_i_* is the concentration of A molecules in the subvolume from which the molecules diffuse.

The rates, i.e., the probabilities per time unit, of all different diffusion and reaction events define a stochastic process. When one event occurs, some of the probabilities for the next event will change. The RDME describes how the probability that the system is in a certain state changes in time. Unfortunately, the RDME can not be solved analytically except for very simple systems [60], and direct numerical integration is not possible due to the vastness of the state space. As an alternative, it is possible to follow a single trajectory of the system by sampling one event at the time [46], and then update the state and the probabilities for the next event depending on what event just occurred. The sampling technique must, however, be exceptionally efficient since the state space typically has several million dimensions. The Next Subvolume Method that we have developed for sampling the reaction–diffusion master equation is described in its own section below.

In this study, we compare the stochastic time evolutions with the corresponding mean-field approximation. The approximation is that the state, i.e., number of molecules, can change continuously and that the state changes with the average rate at each point in time. To see what this means, consider the events that change the number of A molecules in subvolume *i* during the next time period *dt* in a one-dimensional system. Assume that an A molecule can be consumed in a reaction 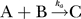 with probability *dt*Ω*k_a_a_i_b_i_* or diffuse away to either of the two neighboring subvolumes with probability *dt*Ω*k_diff_ a_i_*. The number of A molecules in subvolume *i* can also increase if an A molecule diffuses from one of the neighboring subvolumes. The probabilities for these diffusion events are *dt*Ω*k_diff_ a_i−1_* and *dt*Ω*k_diff_ a_i+1_*, respectively. The average change in *n_A,i_* during *dt* is therefore *dn_A,i_* = (−1)*dt*Ω*k_a_a_i_b_i_* + 2(−1)*dt*Ω*k_diff_ a_i_* + (1)*dt*Ω*k_diff_ a_i−1_* + (1)*dt*Ω*k_diff_ a_i+1_*, where the values in parenthesis are the stoichiometries of the events. Dividing by Ω and using *k_diff_* = *D/ℓ* ^2^ *,* we see that the average change in concentration during *dt* is given by *da_i_* = *dn_A,i_*/Ω = *dt*(−*k_a_a_i_b_i_* + *D*(*a_i_*_−1_ − 2*a_i_* + *a_i_*_+1_)/*ℓ* ^2^). Similar expressions can be straightforwardly derived for all species and subvolumes in the system. If *dt* and *ℓ* are small, the expressions may be used as a numerical scheme for evolving the mean-field equations in time. In the limits *ℓ* → 0, *dt* → 0, we get the mean-field equations, for instance ∂*a*(*x,t*)/∂*t* = −*k_a_* *a*(*x,t*)*b*(*x,t*) + *D*∂^2^ *a*(*x,t*)/∂^2^ *x* with *x* as the continuous spatial coordinate.

### MesoRD.

We have developed the MesoRD software [44] to make it easy to sample trajectories corresponding to the RDME of arbitrary reaction networks in complex geometries. This study is the first application of MesoRD to a real biological system. For the deterministic analysis, MesoRD was extended to include numerical integration of mean-field equations. The kinetic model is fed to MesoRD as a Systems Biology Markup Language (SBML) file [61], which contains all information about reactions, reactants, and reaction rates. The standard SBML has been extended to include 3D geometry descriptions using Constructive Solid Geometry (CSG). Both the mean-field and the stochastic solver use the same SBML model description input file, which facilitates direct comparison. MesoRD is free software available at http://mesord.sourceforge.net. The source code is openly distributed under GNU GPL license. The SBML file for the Min system is supplied as supporting material to this article (Datasets S1–S9).

### The Next Subvolume Method.

For stochastic simulations, MesoRD uses the Next Subvolume Method (NSM) essentially as described in [5]. The NSM algorithm is equivalent to the *SSA* algorithm [62,63] and the Next Reaction Method [64] for sampling discrete-state Markov processes in continuous time, but it is adapted to the structure of the RDME, which makes it efficient enough to simulate 3D systems.

In NSM, the rates of all elementary events are summed for each subvolume, and the time of the next event in each subvolume is sampled from their respective exponential distributions. Based on these event times, the subvolumes are ordered in a priority queue (stored as a binary tree). The next reaction or diffusion event occurs in the subvolume that is first in the queue. This event will only change the states, rates, and next event time for maximally two different subvolumes. Therefore, in each iteration, only the one or two queue elements that correspond to the subvolumes with a state change in the last event need to be updated and sorted.

In the NSM, the number of computations scales logarithmically with the number of subvolumes instead of linearly as in the *SSA* algorithm. For large reaction–diffusion problems, the NSM is also several orders of magnitude faster than a direct application of the Next Reaction Method (See the supplementary material of Elf and Ehrenberg [5] for a more detailed description). The NSM is also used in the SmartCell software [65].

The most important algorithmic improvement in MesoRD, as compared to our original formulation of the NSM, is that we now use a hash table to look up reaction rates corresponding to the commonly occurring combinations of reactants per subvolume. Since the number of reactants of each species per subvolume usually is very low (zero, one, or two), the rates for all commonly occurring combinations can be precalculated. Furthermore, in the case that two subvolumes needs to be sorted, the one with the earliest next event time is sorted from the top of the queue.

### Numerical integration of the mean-field equations.

A set of different partial differential equation (PDE) solvers was implemented in MesoRD. All the solvers are based on the method of lines [66]. When the spatial dependency has been removed, the resulting system of ordinary differential equations (ODE) can be solved with a variety of ODE solvers. In this study, the backward differentiation formula (BDF) with two steps was used.

### The Smoluchowski alternative.

As an alternative to the RDME [34–37], stochastic reaction–diffusion may be described in the Smoluchowski formalism [38–42]. In the Smoluchowski formalism, the locations of individual particles are modeled by probability density functions spreading in space over time. Here, reactions are treated as boundary conditions for the partial differential equations that describe the diffusion of the particles. Exact realizations of this stochastic process can be sampled by the Green's Function Reaction Diffusion algorithm (GFRD) [67]. The GFRD algorithm is event driven which makes it highly efficient, because large jumps in time and space can be made when the particles are far apart from each other. Before GFRD, it was impossible to sample exact trajectories of a system with more than two interacting particles described in the Smoluchowski formalism.

Although the GFRD algorithm is a computational breakthrough, it is likely to be too computationally demanding for a direct application to the Min system. However, if one does not need exact interaction information in time and space, there are at least three simulation tools that can be used: Mcell [68], SmolDyn [69], and ChemCell [70]. In these tools, the Brownian motion of all molecules is sampled at appropriate time intervals. Depending on the positions of the molecules in space, it is decided if nearby molecules have reacted or not during the last time interval. MCell, SmolDyn, and ChemCell make this decision in different ways.

As a point of reference to the RDME treatment, one can consider the case in which the time step for the Brownian diffusion is chosen as the mean time between diffusion events in the RDME description, i.e. *ℓ* ^2^/2*D* in one dimension. In this case the root mean square (rms) displacement during the time step equals the length of one subvolume. If, in addition, the reaction probability during this time step is calculated from the local concentration within a radius equal to the rms, the particle-based and RDME-based methods are very similar.

### What about MinD polymerization?

The MinD protein forms polymers in vitro if both ATP and phospholipids are present [17,18]. In vivo 3D image reconstruction shows that MinD polymers form helices along the membrane [71]. An apparent difference between our model and the real system is that we do not account for this polymerization. So why does the model work without polymers? One interpretation could be that the membrane-bound MinD can recruit cytosolic MinD^ATP^ almost equally efficiently in monomer and polymer form and that MinE can hydrolyze MinD^ATP^ anywhere in the polymers. If this is the case, there is no practical difference between our model and a model with polymerized MinD except that the polymerization makes the MinD spread along the membrane, which we account for by slow membrane diffusion. The exact value of the membrane diffusion rate is, however, unimportant as long as it is significantly lower than the cytosolic diffusion rate but not zero, which would lead to unphysiologically high local concentrations.

To justify the membrane diffusion model we have made a control simulation using a more detailed membrane translocation model in which the membrane-associated MinD is immobile unless the local membrane occupancy is higher than ten MinD molecule per 2,500 nm^2^ (one molecule per 15 nm × 15 nm) in which case one MinD molecule is moved to a neighboring, less crowded, location. This translocation model, which approximates the effects of MinD polymerization on the membrane, gives very similar results to the more simple model with a slow diffusion of membrane-bound MinD.

One experimental observation that we believe may require polymerization for quantitative modeling is the stuttering in the shrinkage phase for the MinD zone. This stuttering is observed especially in certain MinE mutants [21,43]. We suggest that stuttering depends on near critical fluctuations [72,73] in the length of MinD polymers. This phenomenon would cause the observed stuttering behavior if the rates of polymerization of MinD and of hydrolysis by MinE were closely balanced in such a way that the average shrinkage speed would be slow although the turnover of monomers was high.

## Supporting Information

Dataset S1 SBML File for Stochastic Simulation of wt Geometry of Length 4.5 μm This file is also deposited in the BioModels Database ID: MODEL5974712823.
(11 KB XML)

Dataset S2 SBML File for Mean-Field Simulation of wt Geometry of Length 4.5 μm (25 KB XML)

Dataset S3 SBML File for Stochastic Simulation of Filamentous Geometry of Length 10.5 μm (11 KB XML)

Dataset S4 SBML File for Mean-Field Simulation of Filamentous Geometry of Length 10.5 μm (25 KB XML)

Dataset S5 SBML File for Stochastic Simulation of Spherical Geometry of Radius 1.5 μm (10 KB XML)

Dataset S6 SBML File for Mean-Field Simulation of Spherical Geometry of Radius 1.5 μm (24 KB XML)

Dataset S7 SBML File for Stochastic Simulation of Filamentous Geometry of Length 15.5 μm (10 KB XML)

Dataset S8 SBML File for Mean-Field Simulation of a PE^−^ Cell of Length 10.5 μm (11 KB XML)

Dataset S9 SBML File for Mean-Field Simulation of a PE^−^ Cell of Length 10.5μm (25 KB XML)

Figure S1 Stochastic Trajectory in a 15-μm Filamentous Cell Starting from a Uniform Distribution of Molecules Membrane-bound MinD molecules are shown in blue and membrane-bound MinDE complexes are shown in red.
(832 KB PDF)

Figure S2 Time-Evolution of Nucleation Processes for Different Numbers of Initiator Molecules The stochastic model with PE^−^ parameters was solved for a box (5 μm × 1 μm × 1 μm), with membrane on the 1-μm × 1-μm side. The simulations were initialized with a membrane occupancy of 1–12 MinD molecules. A total of 100 trajectories were gathered for each number (1–12) of initiator molecules. The probability of nucleation is defined as the fraction of trajectories reaching more than 100 membrane-bound MinD molecules.
(192 KB PDF)

Video S1 Stochastic Simulation of a wt Geometry of Length 4.5 μm Initial conditions as described in the text. Membrane-bound MinD is shown in blue, and MinD in complex with MinE on the membrane is shown in red.
(3.0 MB MOV)

Video S2 Mean-Field Simulation of a wt Geometry of Length 4.5 μm Initial conditions as described in the text. Membrane-bound MinD is shown in blue, and MinD in complex with MinE on the membrane is shown in red.
(644 KB MOV)

Video S3 Stochastic Simulation of a Filamentous Geometry of Length 10.5 μm Initial conditions as described in the text. Membrane-bound MinD is shown in red, and MinD in complex with MinE on the membrane is shown in blue.
(3.0 MB MOV)

Video S4 Mean-Field Simulation of a Filamentous Geometry of Length 10.5 μm Initial conditions as described in the text. Membrane-bound MinD is shown in blue, and MinD in complex with MinE on the membrane is shown in red.
(946 KB MOV)

Video S5 Stochastic Simulation of a Spherical Geometry with Radius 1.5 μm Initial conditions as described in the text. Membrane-bound MinD is shown in blue, and MinD in complex with MinE on the membrane is shown in red.
(2.8 MB MOV)

Video S6 Mean-Field Simulation of a Spherical Geometry with Radius 1.5 μm The simulation is started with most of the MinD molecules bound to the membrane on one side of the cell. Membrane-bound MinD is shown in blue, and MinD in complex with MinE on the membrane is shown in red.
(278 KB MOV)

Video S7 Stochastic Simulation of a Filamentous Geometry of Length 15.5 μm Initial conditions as described in the text. Membrane-bound MinD is shown in blue, and MinD in complex with MinE on the membrane is shown in red.
(2.9 MB MOV)

Video S8 Stochastic Simulation of a PE^−^ Strain of Length 10.5 μm Initial conditions as described in the text. Membrane-bound MinD is shown in blue, and MinD in complex with MinE on the membrane is shown in red.
(242 KB MOV)

Video S9 Mean-Field Simulation of a PE^−^ Strain of Length 10.5 μm Initial conditions as described in the text. Membrane-bound MinD is shown in blue, and MinD in complex with MinE on the membrane is shown in red.
(28 KB MOV)

### Accession Numbers

The Ecogene Database of Escherichia coli Sequence and Function (http://www.ecogene.org) accession numbers for the genes discussed in this paper are as follows: *ftsZ* (EG10347), *minC* (EG10596), and *rodA* (EG10607). The UniProt (http://www.ebi.uniprot.org) accession numbers for the proteins discussed in this paper are as follows: FtsZ (P0A9A6), MinC (P18196), MinD (P0AEZ3), MinE (P0A734), and Soj (P37522). The BioModels Database (http://www.biomodels.org) accession number for the wt model discussed in this paper is: MODEL5974712823.

## Abbreviations

3D: three dimensional
GFRD: Green's Function Reaction Diffusion algorithm
NSM: Next Subvolume Method
PE: phospathedylethanolamide
PE^−^: phospathedylethanolamide deficient
RDME: reaction–diffusion master equation
SBML: Systems Biology Markup Language
wt: wild type
