# Supplementary figures and images for: Noise-Induced Min Phenotypes in E. coli
Source: PLoS Comput Biol. 2006 Jun 30;2(6):e80. doi: 10.1371/journal.pcbi.0020080 (PMC1484588; doi:10.1371/journal.pcbi.0020080)

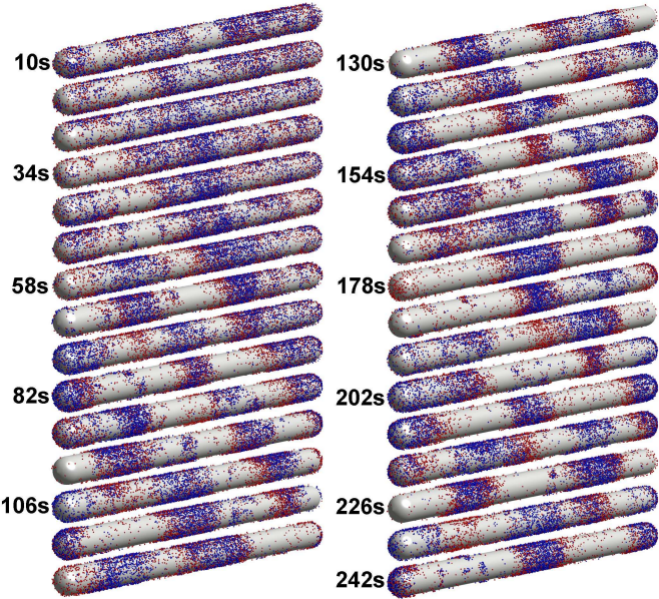

Supplement: Figure S1 — Membrane-bound MinD molecules are shown in blue and membrane-bound MinDE complexes are shown in red. (832 KB PDF) [file pcbi.0020080.sg001.pdf]

Total Number of MinD Molecules in Membrane

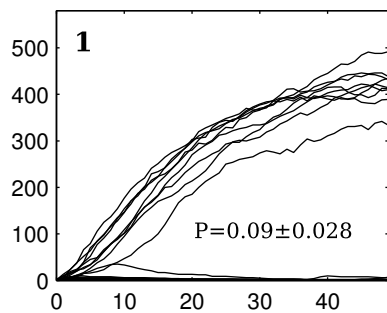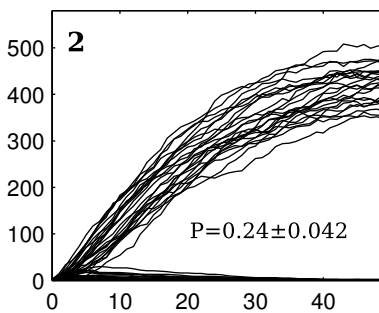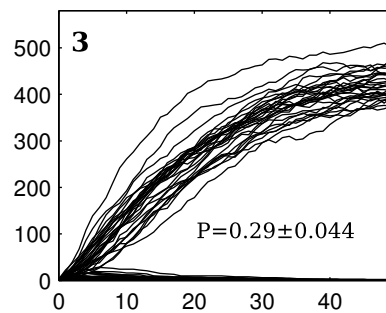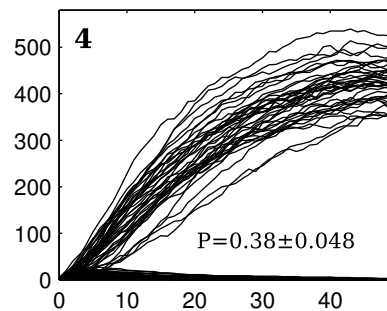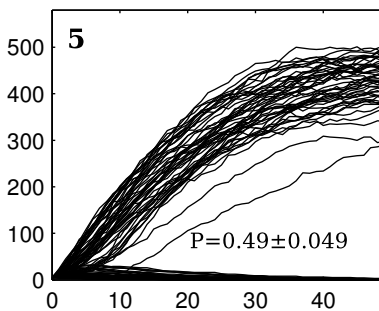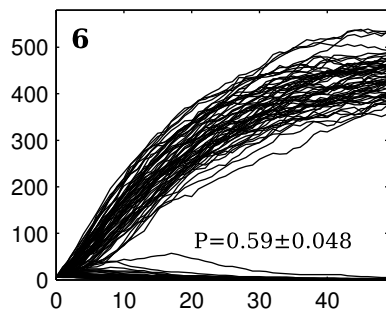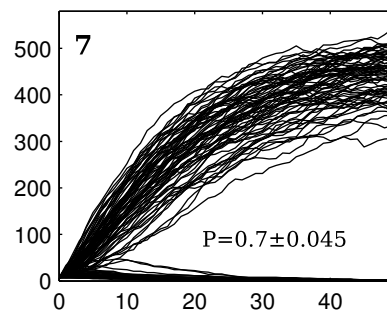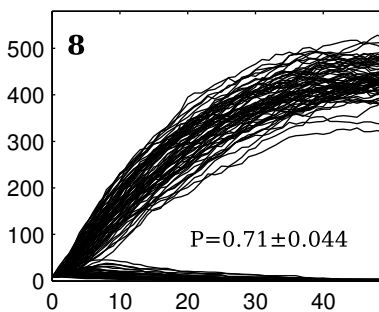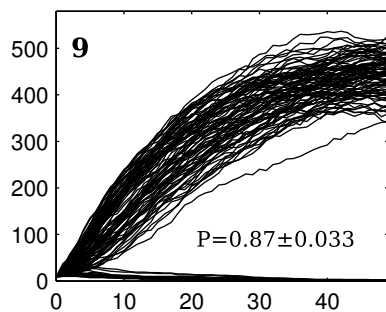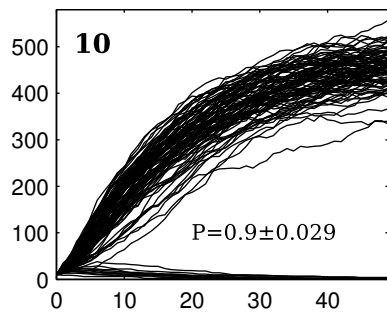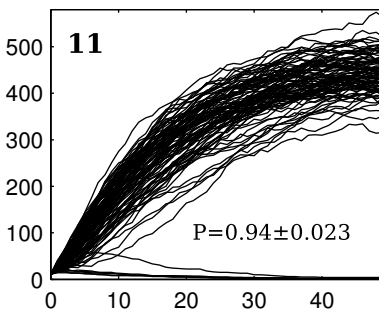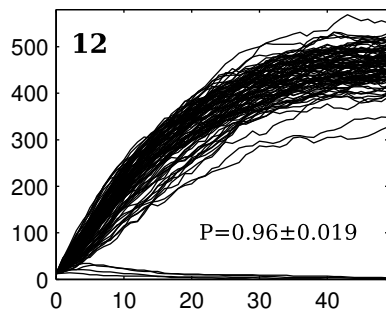

Time (0.1xs)

Supplement: Figure S2 — The stochastic model with PE− parameters was solved for a box (5 μm × 1 μm × 1 μm), with membrane on the 1-μm × 1-μm side. The simulations were initialized with a membrane occupancy of 1–12 MinD molecules. A total of 100 trajectories were gathered for each number (1–12) of initiator molecules. The probability of nucleation is defined as the fraction of trajectories reaching more than 100 membrane-bound MinD molecules. (192 KB PDF) [file pcbi.0020080.sg002.pdf]
